# Tailoring the structural and functional properties of spray-pyrolyzed Ga_2_O_3_ thin films via Al doping

**DOI:** 10.3762/bjnano.17.91

**Published:** 2026-09-25

**Authors:** Vadim Morari, Geanina V Mihai, Abdulkarim Alshibani, Emil V Rusu, Veaceslav V Ursaki, Marius Enachescu

**Affiliations:** 1 D. Ghitu Institute of Electronic Engineering and Nanotechnologies, Technical University of Moldova, Academiei str. 3/3, Chisinau MD-2028, Republic of Moldova; 2 Center for Surface Science and NanoTechnology, University Politehnica of Bucharest, 060042-Bucharest, Romania; 3 National Center for Materials Study and Testing, Technical University of Moldova, Chisinau 2004, Republic of Moldova; 4 Academy of Sciences of Moldova, Bv. Stefan cel Mare 1, Chisinau 2001, Republic of Moldova; 5 Academy of Romanian Scientists, Str Ilfov 3, Bucharest, 030167, Romania

**Keywords:** atomic force microscopy (AFM), Al-doped, bandgap, Ga_2_O_3_, *I*–*V* characteristics, scanning electron microscopy (SEM), X-ray diffraction (XRD)

## Abstract

Undoped and Al-doped α-Ga_2_O_3_ thin films (1–3 vol % of Al in precursor solution) were successfully deposited on Si substrates by spray pyrolysis at 500 °C and systematically investigated in terms of morphological, structural, compositional, optical, and electrical properties. Atomic force microscopy analysis revealed that moderate Al incorporation significantly improves surface morphology, reducing the root mean square roughness from 2.0 nm (undoped) to a minimum of 1.25 nm at 2 vol % Al, followed by a slight increase at 3 vol % Al due to possible lattice strain and compositional inhomogeneity. Cross-sectional scanning electron microscopy images confirmed the formation of dense, crack-free, and well-adhered films with thicknesses ranging from 445 to 540 nm, indicating that Al incorporation affects growth kinetics. Energy-dispersive X-ray analysis verified successful Al incorporation and slight compositional variations, suggesting modifications in defect chemistry. X-ray diffraction results demonstrated that all films crystallize in the α-Ga_2_O_3_ phase with a corundum-type structure, while Al doping significantly influences crystal orientation, crystallite size, and lattice strain without altering the phase. Optical measurements revealed high transparency (≈90%) in the visible region for all samples. The optical bandgap increased slightly from 4.82 eV (undoped) to 4.87 eV (3 vol % Al), indicating successful substitutional incorporation of Al^3+^ and tunable optical properties. Electrical measurements showed linear current–voltage characteristics, confirming ohmic conduction. The resistance decreased from 17.32 MΩ (undoped) to 0.47 MΩ (3 vol % Al), demonstrating enhanced conductivity while preserving the semiconducting nature of the material. Overall, controlled Al doping effectively tailors the microstructural, optical, and electrical properties of α-Ga_2_O_3_ thin films, with 2 vol % Al identified as the optimal concentration for achieving improved surface morphology and balanced functional performance.

## Introduction

Gallium oxide (Ga_2_O_3_) is an ultrawide-bandgap semiconductor (*E*_g_ = 4.5–5.0 eV) [1] that has attracted considerable attention for next-generation power electronics, deep-UV optoelectronics, and high-temperature sensing applications [2–5]. Owing to its large breakdown electric field, high chemical and thermal stability, and optical transparency in the ultraviolet region, Ga_2_O_3_ is considered a strong candidate for high-power and high-frequency devices [6]. Among its polymorphs, the β-phase (β-Ga_2_O_3_) is the most thermodynamically stable and widely investigated for device applications [7].

The electrical properties of Ga_2_O_3_ can be tailored through controlled doping to modify the free carrier concentration and, consequently, the electrical conductivity. n-Type conductivity is commonly achieved by doping with donor elements such as Nb, Sn, Ge or Si, which introduce shallow donor levels near the conduction band [8–11]. The choice of dopant and its concentration strongly influence not only the electrical conductivity but also the structural, optical, and morphological properties of the material. In particular, doping with Al has been explored to tune the bandgap and modify transport properties through the formation of (Al*_x_*Ga_1−_*_x_*)_2_O_3_ solid solutions [12–13]. Controlled Al incorporation also enables effective tuning of structural properties of (Al*_x_*Ga_1−_*_x_*)_2_O_3_ thin films, where moderate doping improves crystalline quality and interface stability, while excessive Al content induces lattice strain and defect accumulation, ultimately influencing the electronic performance of the heterostructure. Al doping has been widely investigated as an effective strategy to modify the structural, optical, and electrical properties of Ga_2_O_3_ thin films. Previous studies have demonstrated that the incorporation of Al into the Ga_2_O_3_ lattice can influence the carrier concentration, optical bandgap, and defect-related properties, depending on deposition technique and doping level [14]. The incorporation of Al^3+^ ions into the Ga_2_O_3_ lattice is facilitated by the similarity in valence state between Al^3+^ and Ga^3+^ ions. However, their ionic radii are different, with Al^3+^ (approximately 0.535 Å) being smaller than Ga^3+^ (approximately 0.62 Å) for tetrahedral coordination. This difference facilitates the incorporation of Al ions into the Ga_2_O_3_ lattice, on the one hand. However, the partial substitution of Ga^3+^ by smaller Al^3+^ ions can induce lattice contraction and local strain within the Ga_2_O_3_ structure, on the other hand. Such structural modifications may influence defect formation, particularly oxygen vacancy concentration, which can affect the electrical and optical characteristics of the films [12,14].

Various techniques have been employed for the fabrication of α-Ga_2_O_3_ thin films, including sol–gel processing [15], magnetron sputtering [16], chemical vapor deposition (CVD) [17], molecular beam epitaxy (MBE) [18], pulsed laser deposition (PLD) [19], atomic layer deposition (ALD) [20] and spray pyrolysis [21]. Among these, spray pyrolysis is an attractive and cost-effective method due to its simplicity, scalability, and suitability for large-area deposition. The technique allows for good control over film thickness and composition by adjusting parameters such as precursor concentration, substrate temperature, and spray rate [22–23].

Gallium oxide exists in several polymorphic forms, including α-, β-, γ-, δ-, and ε-Ga_2_O_3_ phases. Among these, the monoclinic β-Ga_2_O_3_ phase is thermodynamically the most stable and has been extensively studied for high-power electronics and ultraviolet optoelectronic devices due to its ultrawide bandgap (≈4.8 eV) and high breakdown field. The metastable α-Ga_2_O_3_ phase, which exhibits a corundum-type structure similar to that of α-Al_2_O_3_, has attracted increasing attention because of the potential for high-power devices and compatibility with heterostructure engineering. However, the α-phase is thermodynamically less stable and can transform into more stable phases depending on growth conditions and thermal treatment [24].

In the present study, Al-doped α-Ga_2_O_3_ thin films (1–3 vol % of Al in precursor solution) were prepared by spray pyrolysis in order to investigate the influence of Al incorporation on the structural and electrical properties. Particular attention was given to the correlation between Al doping level and the resulting changes in crystallinity, microstructure, and electrical conductivity.

## Results and Discussion

**Figure 1:**
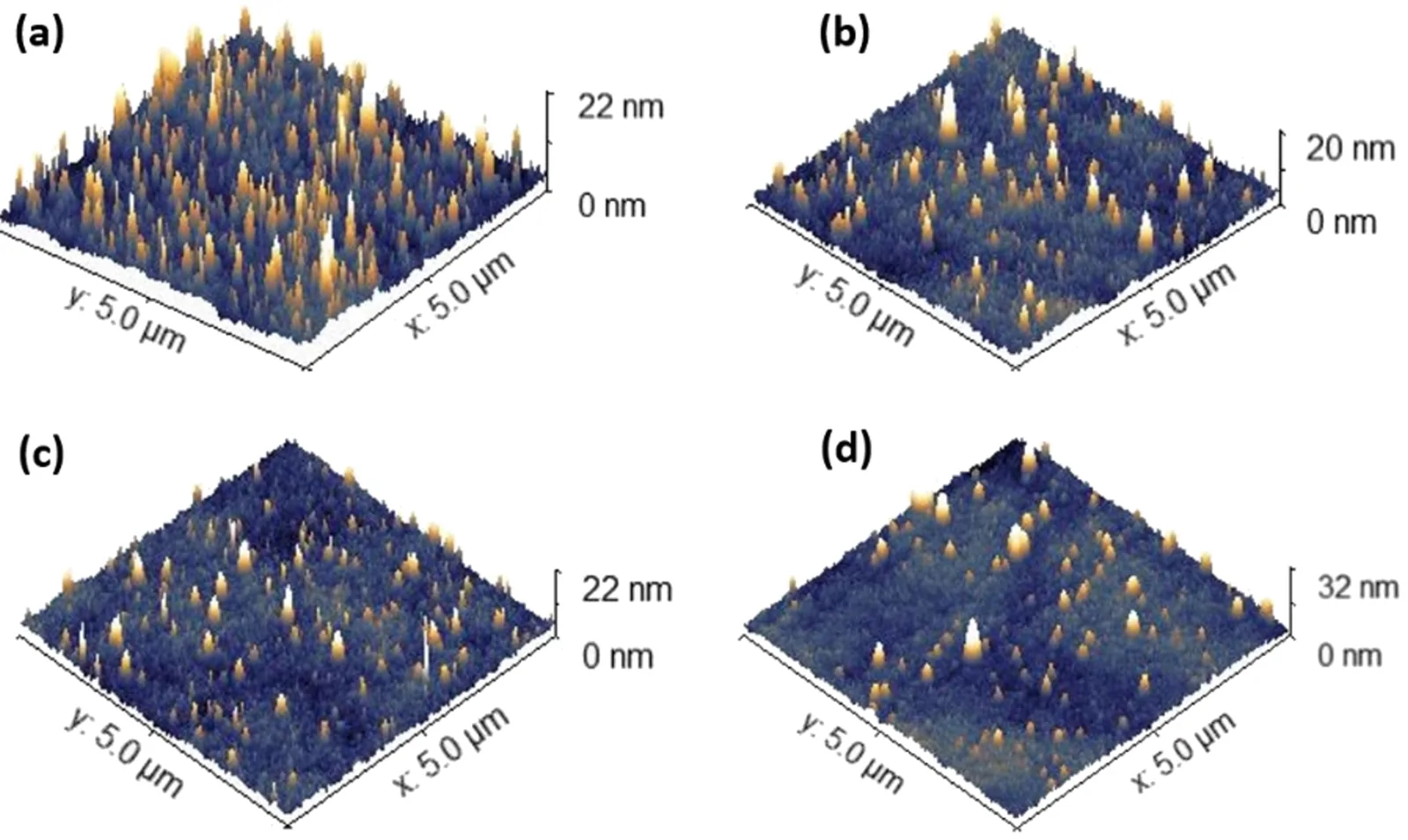
AFM topography (5 × 5 µm^2^) of undoped and Al-doped α-Ga_2_O_3_ films as follows: 0 vol % (a), 1 vol % (b), 2 vol % (c), and 3 vol % (d).

Surface topography imaging and quantitative surface roughness analysis of the undoped and Al-doped α-Ga_2_O_3_ thin films were carried out using atomic force microscopy (AFM) over a scan area of 5 × 5 μm^2^. The corresponding three-dimensional AFM images are presented in Figure 1. The undoped α-Ga_2_O_3_ film exhibits a relatively rough surface with a root mean square (RMS) roughness of 2.0 nm and an average roughness (*R*_a_) of 1.35 nm. The surface consists of densely distributed sharp protrusions, indicating non-uniform grain growth and significant surface irregularities. Upon incorporation of 1 vol % Al, the surface roughness decreases considerably (RMS = 1.42 nm, *R*_a_ = 0.92 nm). A further reduction in roughness is observed for the 2 vol % Al-doped α-Ga_2_O_3_ film, which exhibits the lowest RMS (1.25 nm) and *R*_a_ (0.81 nm) values among all samples. The AFM image reveals a more homogeneous grain distribution with reduced peak density, suggesting improved surface diffusion and enhanced nucleation control during film growth [25]. However, increasing the Al concentration to 3 vol % results in an increase in surface roughness (RMS = 1.81 nm, *R*_a_ = 1.09 nm). The surface morphology shows the reappearance of larger protrusions, indicating that excessive Al incorporation may introduce lattice strain, defect formation, or compositional inhomogeneity. This deterioration in surface smoothness at higher doping concentration may be attributed to clustering effects, which disturb the growth kinetics.

Overall, the roughness trend follows the relationship


[1] $$\begin{array}{c} {\text{Ga}}_{2}{\text{O}}_{3}\to \text{decreases }\left(1\text{ vol }\%\text{ Al}\right)\\ \to \text{minimum }\left(2\text{ vol }\%\text{ Al}\right)\\ \to \text{increases }\left(3\text{ vol }\%\text{ Al}\right). \end{array}$$

These results indicate that moderate Al doping (2 vol %) optimizes the surface morphology, producing smoother and more uniform films. Such reduced surface roughness is advantageous for optoelectronic and electronic device applications, as it minimizes interface scattering, improves contact formation, and enhances overall device performance. The extracted roughness parameters are summarized in Table 1.

**Table 1:** The roughness parameters of the undoped and Al-doped α-Ga_2_O_3_ films.

Samples	Scan size (µm^2^)	RMS (nm)	*R*_a_ (nm)

α-Ga_2_O_3_	5 × 5	2.0	1.35
α-Ga_2_O_3_:Al (1 vol %)		1.42	0.92
α-Ga_2_O_3_:Al (2 vol %)		1.25	0.81
α-Ga_2_O_3_:Al (3 vol %)		1.81	1.09

**Figure 2:**
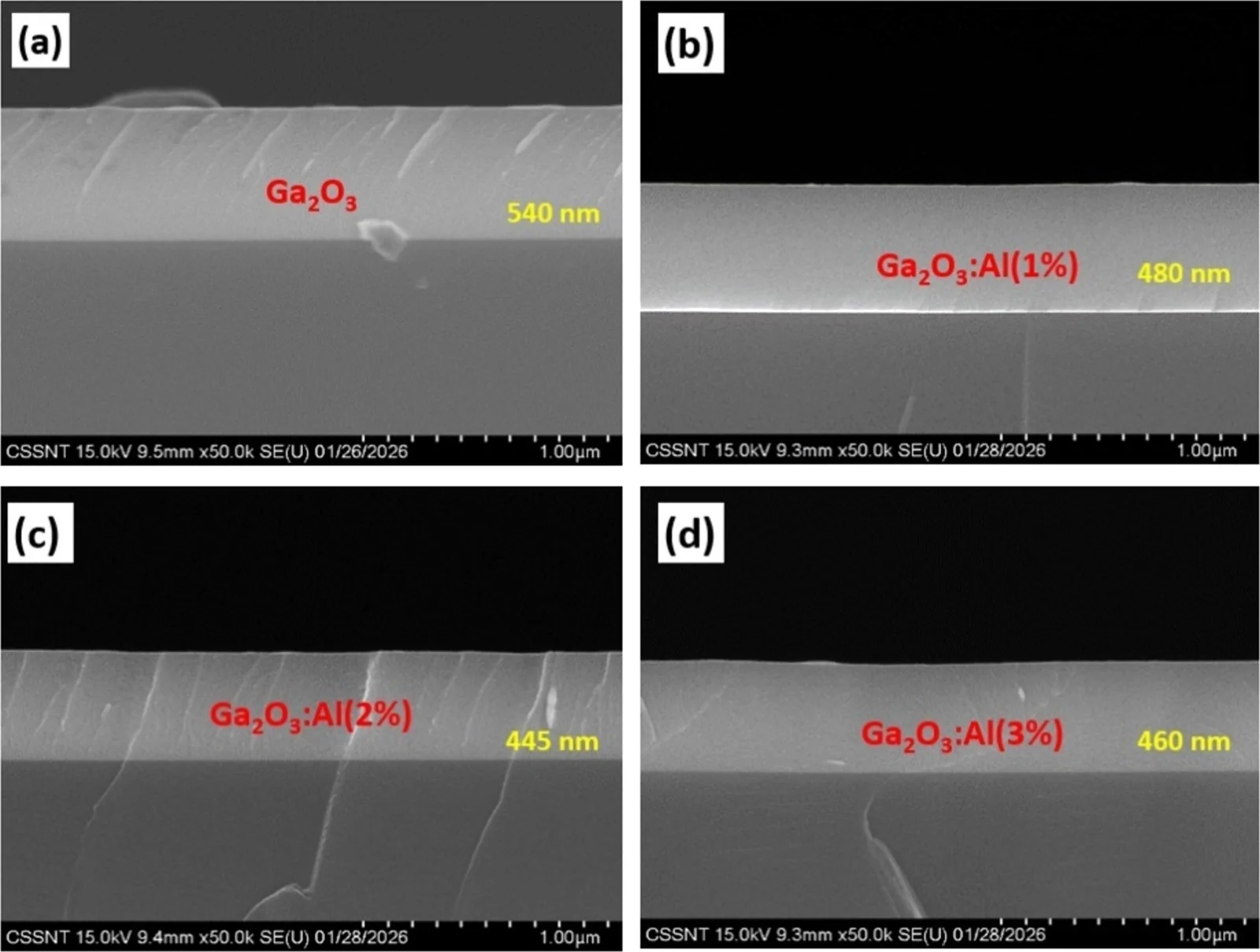
Cross-sectional SEM micrographs of α-Ga_2_O_3_ thin films with varying Al doping concentrations: (a) undoped, (b) 1 vol %, (c) 2 vol %, and (d) 3 vol % Al incorporation.

Cross-sectional SEM micrographs of the undoped and Al-doped α-Ga_2_O_3_ thin films are presented in Figure 2. All samples exhibit a continuous and dense morphology with a well-defined film/substrate interface, indicating good adhesion and uniform growth. No significant cracks, delamination, or voids are observed, confirming the formation of compact oxide layers [26].

The thickness of the undoped α-Ga_2_O_3_ film was estimated to be approximately 540 nm (Figure 2a). In contrast, the Al-doped α-Ga_2_O_3_ films show a slight reduction in thickness. The thickness values were found to be around 480 nm for α-Ga_2_O_3_:Al (1 vol %) (Figure 2b), 445 nm for α-Ga_2_O_3_:Al (2 vol %) (Figure 2c), and 460 nm for α-Ga_2_O_3_:Al (3 vol %) (Figure 2d). This thickness variation suggests that the incorporation of Al affects the film growth kinetics, likely modifying the nucleation rate and deposition efficiency. Overall, the SEM results confirm the successful deposition of homogeneous α-Ga_2_O_3_-based thin films, while Al incorporation leads to noticeable changes in film thickness, reflecting its influence on the growth mechanism.

The chemical composition of all thin films was investigated using EDX analysis, and the results are presented in Table 2. The obtained results confirm the presence of Ga and O as the main constituents in all samples, while Al is detected only in the Al-doped α-Ga_2_O_3_ films, demonstrating the successful incorporation of Al into the α-Ga_2_O_3_ matrix.

**Table 2:** EDX analysis of the undoped and Al-doped α-Ga_2_O_3_ films.

Samples	O (atom %)	Ga (atom %)	Al (atom %)

α-Ga_2_O_3_	55.20	44.80	0.00
α-Ga_2_O_3_:Al (1 vol %)	53.79	42.97	3.24
α-Ga_2_O_3_:Al (2 vol %)	51.97	42.75	5.28
α-Ga_2_O_3_:Al (3 vol %)	51.80	42.50	5.70

For the undoped α-Ga_2_O_3_ film, the atomic concentrations were 55.20 atom % O and 44.80 atom % Ga, with no detectable Al content. After Al doping, the Al concentration increased systematically with the dopant level, reaching 3.24 atom % for α-Ga_2_O_3_:Al (1 vol %), 5.28 atom % for α-Ga_2_O_3_:Al (2 vol %), and 5.70 atom % for α-Ga_2_O_3_:Al (3 vol %). At the same time, a slight decrease in the oxygen content was observed, from 55.20 atom % (undoped) to 51.80–53.79 atom % for the Al-doped α-Ga_2_O_3_ films. It should be noted that the Al content in the precursor solution (vol %) represents the nominal doping level, whereas the actual Al incorporation in the films was determined by EDX analysis and is expressed in atomic percentage (atom %). The measured Al concentration in the films is higher than the nominal value introduced in the precursor solution. A difference between the nominal dopant concentration in the precursor solution and the dopant concentration determined in the deposited films by EDX has also been observed in our previous studies on Mg-doped ZnO, as well as in related studies reported in the literature [27–28]. Although these studies concern different material systems and dopant species, they support the general observation that the nominal precursor composition does not necessarily correspond directly to the elemental composition of the deposited film. This is particularly relevant for spray-pyrolysis deposition, where the final film composition depends on the precursor chemistry and the processes occurring during droplet transport, solvent evaporation, precursor decomposition, and film formation. In addition, the oxygen content determined by EDX is lower than the stoichiometric value expected for α-Ga_2_O_3_. This difference can be attributed to the inherent limitations of EDX analysis for accurately quantifying light elements such as oxygen. Nevertheless, the variation in elemental composition may also indicate that Al incorporation affects the local chemical environment and defect structure of the films, particularly the concentration of oxygen vacancies, which can influence their electrical and optical properties.

**Figure 3:**
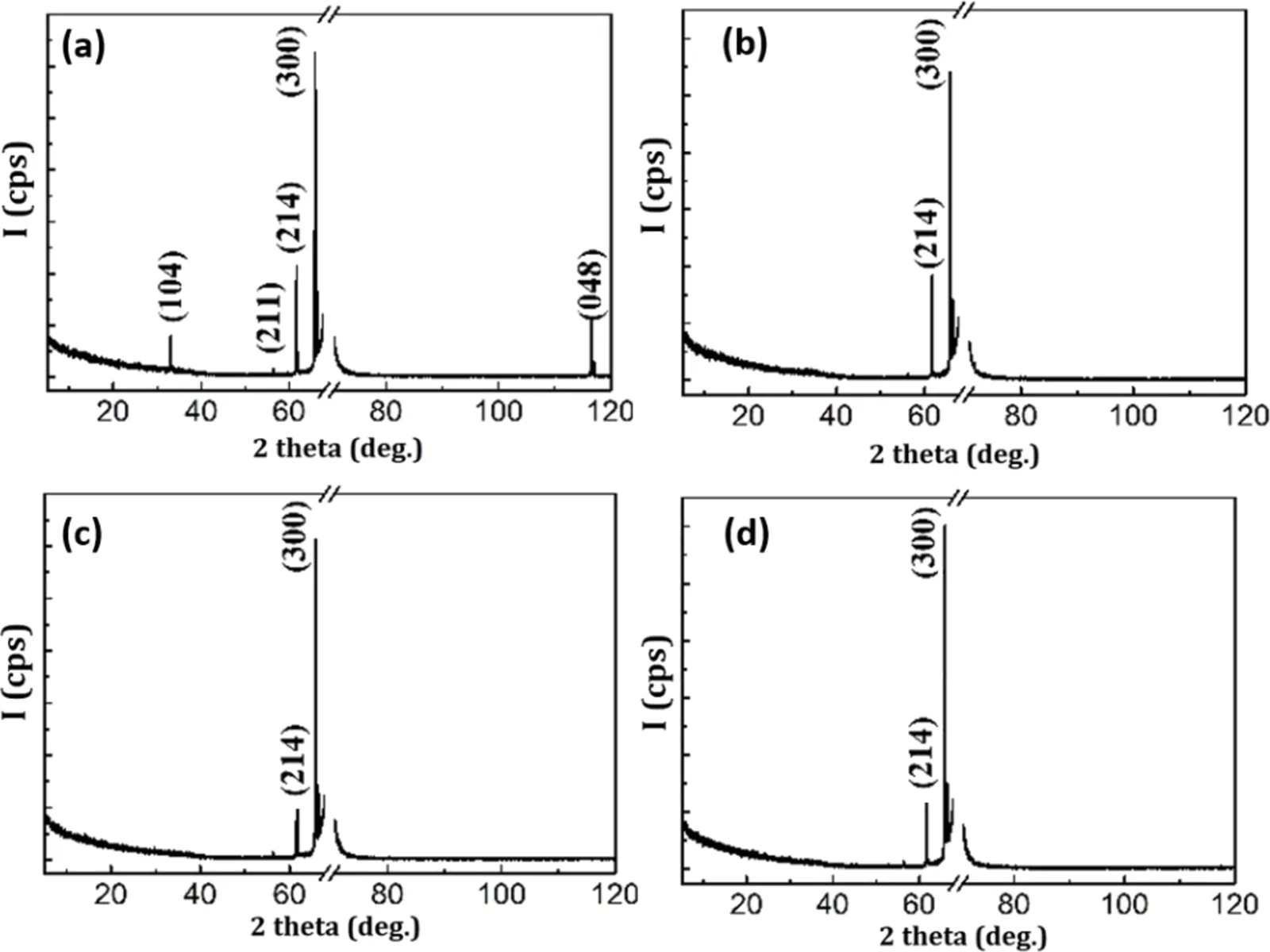
XRD patterns of α-Ga_2_O_3_ thin films with varying Al doping concentrations: (a) undoped (0 vol %), (b) 1 vol %, (c) 2 vol %, and (d) 3 vol % Al incorporation.

The structural properties of the obtained thin films were investigated using X-ray diffraction (XRD) (Figure 3). The undoped α-Ga_2_O_3_ film exhibits diffraction peaks corresponding to the (104), (211), (214), (300), and (048) planes, indicating a polycrystalline nature with multiple crystallographic orientations. The intense diffraction peak observed at approximately 69° corresponds to the Si(400) substrate reflection. Due to its significantly higher intensity compared with the diffraction peaks of the α-Ga_2_O_3_ films, this peak was partially truncated in the presented XRD pattern to improve the visualization of the film-related reflections. The lattice parameters of α-Ga_2_O_3_ were taken from the reference crystal structure data reported in the Pearson’s Crystal Data database (data sheet no. 1812181), with *a* = *b* = 0.49825 nm, *c* = 1.3433 nm, α = β = 90°, and γ = 120°. These reference crystallographic parameters were used for phase identification by matching the experimental XRD diffraction peaks with the corresponding crystallographic data. The good agreement between the experimental diffraction peaks and the reference data confirms the formation of the α-Ga_2_O_3_ phase with a corundum-type hexagonal structure.

Crystallite sizes were calculated via the Scherrer equation. The average crystallite size (*D*) was estimated from the XRD patterns using the Scherrer equation (Equation 2):


[2] $$D=\frac{K\lambda }{\beta \cos\theta },$$

where *D* is the mean crystallite size, *K* is the Scherrer constant (typically taken as 0.9), λ is the wavelength of the Cu Kα radiation (1.5406 Å), β is the full width at half maximum (FWHM) of the diffraction peak (in radians), and θ is the Bragg diffraction angle. The lattice strain (ε) was determined from the peak broadening using the following relation (Equation 3), according to the methodology presented in [29]:


[3] $$\varepsilon =\frac{\beta }{4\tan\theta }.$$

The lattice strain is a dimensionless quantity and is reported as ε × 10^−3^ for convenience.

For the undoped film, the (104) plane exhibits the narrowest peak (0.0734°), corresponding to the largest crystallite size of approximately 109.7 nm and the lowest lattice strain, indicating the highest crystalline quality. In contrast, the (214) and (300) planes show broader diffraction peaks, corresponding to smaller crystallite sizes ranging from 53.23 and 52.27 nm and lattice strain values of approximately (1.26–1.21) × 10^−3^, reflecting a higher degree of lattice distortion. These results suggest that the undoped film possesses well-developed crystallites preferentially oriented along the (104) plane, whereas the remaining planes exhibit moderate structural disorder.

Al doping induces notable changes in both crystallinity and crystal texture. For the α-Ga_2_O_3_:Al (1 vol %) film, only the (214) and (300) reflections are observed, corresponding to crystallite sizes of 53.72 and 52.54 nm with lattice strain values of 1.24 × 10^−3^ and 1.21 × 10^−3^, respectively. These results indicate that the incorporation of Al^3+^ slightly modifies the crystal lattice while preserving the overall crystallinity. In the α-Ga_2_O_3_:Al (2 vol %) film, the (214) and (300) reflections correspond to crystallite sizes of 54.60 and 59.29 nm, with lattice strain values of 1.23 × 10^−3^ and 1.13 × 10^−3^, respectively. The decrease in lattice strain for the (300) plane suggests partial relaxation of the crystal lattice despite the increased dopant concentration. For the α-Ga_2_O_3_:Al (3 vol %) film, the (214) and (300) planes exhibit crystallite sizes of 53.09 and 54.44 nm, accompanied by lattice strain values of 1.26 × 10^−3^ and 1.17 × 10^−3^, indicating slight lattice distortion associated with Al incorporation while maintaining the α-Ga_2_O_3_ crystal structure.

The results indicate that Al incorporation in the range of 1–3% does not alter the α-Ga_2_O_3_ phase but significantly influences the preferred crystal orientation, crystallite size, and lattice strain. The observed variations in crystallite size and microstrain confirm that substitution of Ga^3+^ by the smaller Al^3+^ ions induce local lattice distortion, leading to subtle microstructural modifications without changing the crystal phase. All calculated structural parameters, including the crystallite size (*D*) and lattice strain (ε), are summarized in Table 3.

**Table 3:** Structural parameters of undoped and Al-doped α-Ga_2_O_3_ thin films obtained from XRD analysis.

Sample	Miller indices (h k l)	2-theta (°)	*d* (Å)	FWHM (°)	Crystallite size (*D*), nm	Lattice strain (ε × 10^−3^)

Ga_2_O_3_	(214)	61.66	1.50428	0.1727	53.23	1.26
Ga_2_O_3_	(300)	65.85	1.41835	0.1812	52.27	1.21
Ga_2_O_3_:Al (1 vol %)	(214)	61.67	1.50406	0.171	53.72	1.24
Ga_2_O_3_:Al (1 vol %)	(300)	65.86	1.41816	0.1804	52.54	1.21
Ga_2_O_3_:Al (2 vol %)	(214)	61.65	1.5045	0.1693	54.60	1.23
Ga_2_O_3_:Al (2 vol %)	(300)	62.01	1.49662	0.1562	59.29	1.13
Ga_2_O_3_:Al (3 vol %)	(214)	61.67	1.50406	0.174	53.09	1.26
Ga_2_O_3_:Al (3 vol %)	(300)	65.84	1.41854	0.174	54.44	1.17

These structural modifications are expected to influence the optoelectronic behavior and UV photodetection performance of the films, highlighting the importance of controlled Al doping for tailoring material properties. It is known that the chemical nature of the doping element influences the crystallographic structure of the resulting film. Doping of Ga_2_O_3_ with In_2_O_3_ [22,30] or Sc_2_O_3_ [31] leads predominantly to the monoclinic β-phase, while doping with Fe_2_O_3_ [32] or Ti [33] results in the rhombohedral corundum α-phase. Doping with Al leads also predominantly to the corundum α-phase [34]. However, both α- and β-phase can be formed in (Al*_x_*Ga_1−_*_x_*)_2_O_3_ alloys [35]. Furthermore, an orthorhombic k-phase is produced by doping Ga_2_O_3_ with both In_2_O_3_ and Al_2_O_3_ [36–37].

**Figure 4:**
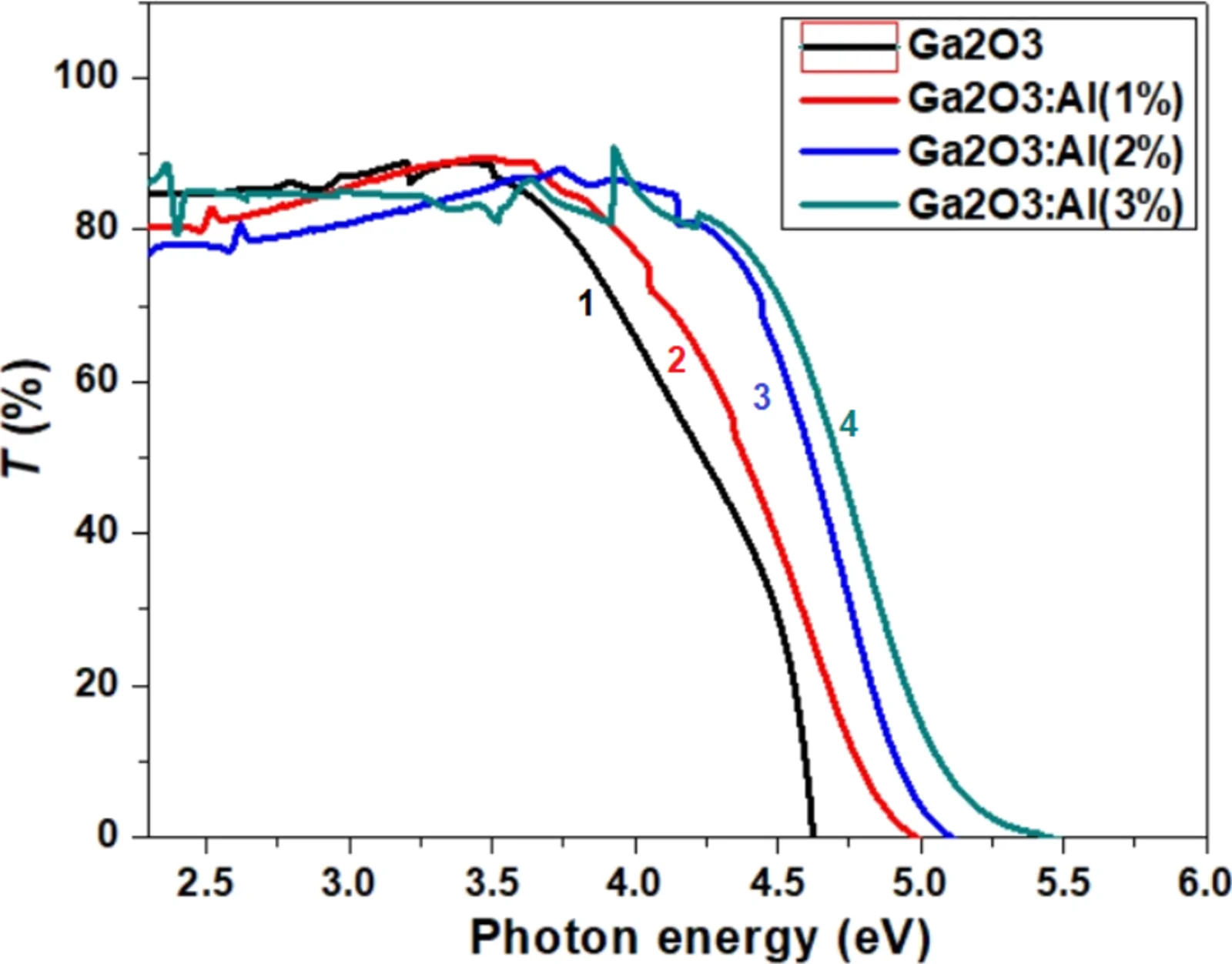
Optical transmittance of α-Ga_2_O_3_ thin films with varying Al doping concentrations: (1) undoped (0 vol %), (2) 1 vol %, (3) 2 vol %, and (4) 3 vol % Al incorporation.

As this study shows, Al doping is an effective strategy for improving the surface smoothness of α-Ga_2_O_3_ thin films compared with the other doping approaches investigated. In addition, this opens up prospects for targeted modification of bandgap width and electrical properties of films. The low processing temperature in spray pyrolysis (400–500 °C) is also an advantage as high processing temperatures of α-Ga_2_O_3_ (800–900 °C) leads to an increase in film roughness by an order of magnitude [21–22]. The optical transmittance spectra of undoped and Al-doped α-Ga_2_O_3_ thin films are presented in Figure 4.

The optical transmittance spectra reveal that all deposited films exhibit high transparency in the visible region. The average transmittance reaches values close to 90% for both undoped and Al-doped α-Ga_2_O_3_ samples. The consistently high transparency across all compositions indicates good optical quality and low light scattering within the films. Furthermore, the incorporation of Al up to 3 vol % does not significantly affect the overall transparency, confirming that low-level doping does not introduce additional absorption centers in the visible range. The small fluctuations observed in the transmittance spectra are mainly attributed to instrumental noise, light scattering effects related to surface roughness, and thin-film interference phenomena. These variations do not significantly influence the determination of the optical bandgap energy (*E*_g_), and the overall optical behavior of the films remains unaffected.

**Figure 5:**
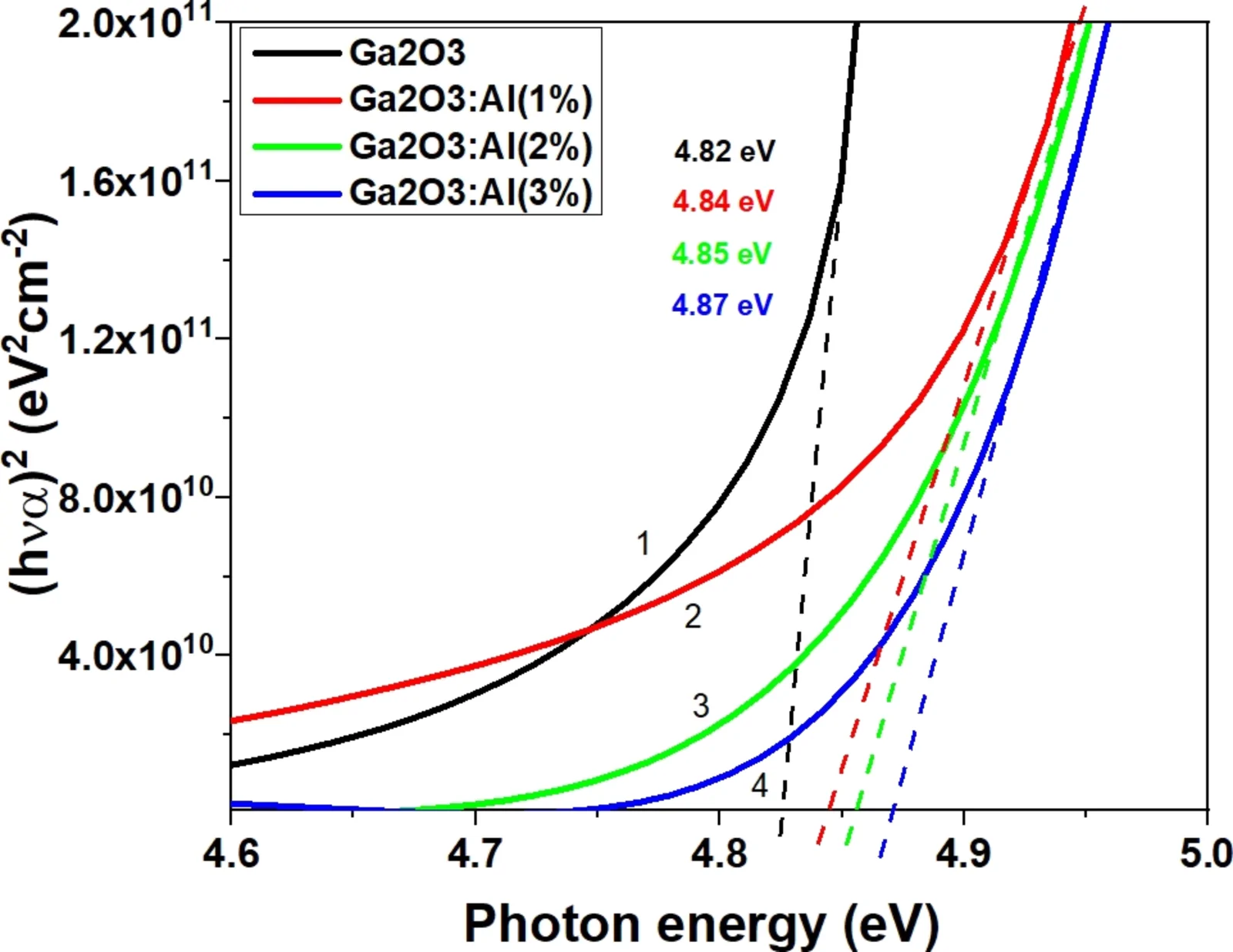
Tauc plots used for bandgap energy determination of α-Ga_2_O_3_ thin films with varying Al doping concentrations: (1) undoped (0 vol %), (2) 1 vol %, (3) 2 vol %, and (4) 3 vol % Al incorporation.

The optical bandgap energy of the undoped and Al-doped α-Ga_2_O_3_ thin films was determined using the Tauc relation for a direct allowed transition, by plotting (α*h*ν)^2^ as a function of photon energy (*h*ν). The extrapolation of the linear region of the (α*h*ν)^2^ curves to 0 yields the optical bandgap values shown in Figure 5.

The undoped α-Ga_2_O_3_ film exhibits a bandgap of 4.82 eV. Upon Al incorporation, a gradual increase in *E*_g_ is observed, reaching 4.84, 4.85, and 4.87 eV for 1, 2, and 3 vol % Al doping, respectively. The systematic widening of the bandgap with increasing Al content confirms its successful incorporation in the solid solution. The increase in *E*_g_ can be attributed to the higher bond energy of Al–O compared to Ga–O, which shifts the conduction band minimum toward higher energies. The nearly linear variation of the bandgap with Al concentration suggests uniform substitution of Ga^3+^ by Al^3+^ within the lattice. Furthermore, the absence of additional absorption features indicates that no secondary phases were formed within the doping range investigated. The relatively small but consistent bandgap increase (≈0.05 eV from 0 to 3% Al) is characteristic of low-level doping and indicates that the optical properties can be finely tuned through controlled Al incorporation.

**Figure 6:**
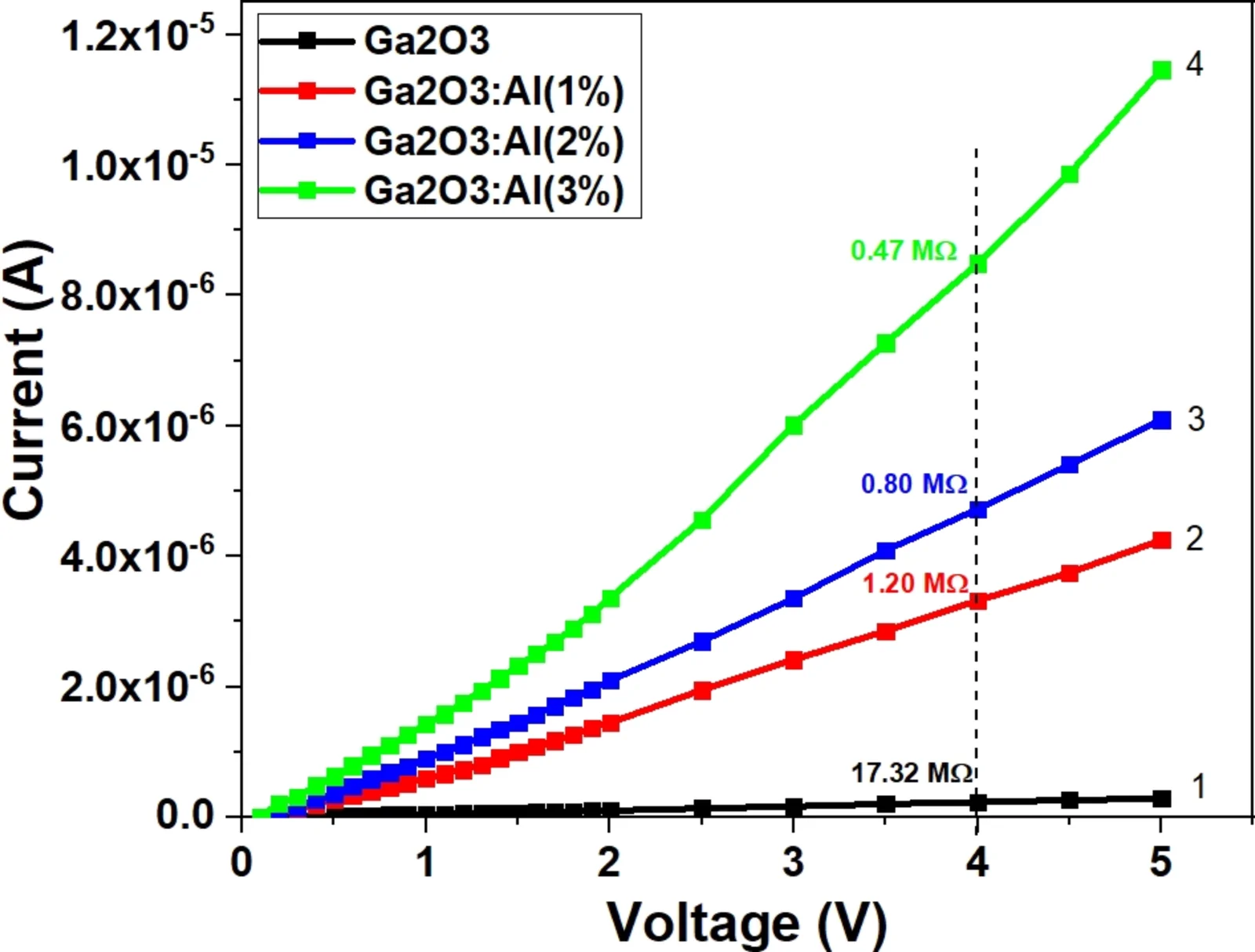
The dark current–voltage (*I*–*V*) characteristics of α-Ga_2_O_3_ thin films with varying Al doping concentrations: (1) undoped (0 vol %), (2) 1 vol %, (3) 2 vol %, and (4) 3 vol % Al incorporation.

Figure 6 presents the current–voltage (*I*–*V*) characteristics of undoped and Al-doped α-Ga_2_O_3_ films with Al concentrations of 1, 2, and 3 vol %. A linear *I*–*V* dependence is observed within the 0–5 V range for all investigated samples, indicating ohmic behavior and confirming the formation of good ohmic contacts. The dominant conduction mechanism in this voltage range is therefore ohmic conduction.

Compared to the undoped sample, the Al-doped samples exhibit a significant increase in current at the same applied voltage, demonstrating a substantial enhancement in electrical conductivity. The resistances of the films were determined using Ohm’s law (*R* = *V*/*I*), based on the linear *I*–*V* characteristics, by evaluating the *V*/*I* ratio at 4 V. The calculated resistance values are 17.32 MΩ for pure α-Ga_2_O_3_, 1.20 MΩ for α-Ga_2_O_3_:Al (1 vol %), 0.80 MΩ for α-Ga_2_O_3_:Al (2 vol %), and 0.47 MΩ for α-Ga_2_O_3_:Al (3 vol %). The progressive decrease in resistance with increasing Al concentration clearly highlights the role of the dopant in modifying the electrical transport properties. The resistance decreases by approximately one order of magnitude at 1 vol % Al doping and by nearly 37 times at 3 vol % Al doping compared to the undoped sample. This behavior suggests an increase in charge carrier concentration and improved carrier mobility. The incorporation of Al ions into the α-Ga_2_O_3_ lattice may introduce donor-like states or reduce compensating defects, thereby facilitating charge transport.

However, it should be emphasized that undoped α-Ga_2_O_3_ thin films are intrinsically highly resistive materials. Although Al doping significantly reduces the resistance, the resulting values remain in the megaohm range, indicating that the films still preserve relatively high resistivity. Therefore, while Al incorporation effectively enhances electrical conductivity, it does not lead to extremely low resistance values, and the material retains its semiconducting character. Al doping is an effective approach for tailoring the electrical properties of α-Ga_2_O_3_. The results demonstrate a systematic increase in conductivity with increasing dopant concentration, while maintaining relatively high resistance values characteristic of wide-bandgap semiconductor materials.

## Conclusion

The results of this study demonstrate that controlled Al incorporation (1–3 vol % in the precursor solution) significantly influences the morphological, structural, optical, and electrical properties of Ga_2_O_3_ thin films, while preserving the α-Ga_2_O_3_ phase and high optical transparency.

Topological analysis revealed that moderate Al doping improves the surface uniformity of the films, with the RMS roughness decreasing from 2.0 nm for the undoped film to a minimum value of 1.25 nm for the 2 vol % Al-doped sample. This behavior can be associated with improved nucleation and surface diffusion processes during film growth. Additionally, the cross-sectional SEM analysis showed a decrease in film thickness from 540 nm for the undoped film to 445 nm for the 2 vol % Al-doped film, which may also contribute to the observed reduction in surface roughness. However, further increasing the Al concentration to 3 vol % resulted in a partial increase in surface roughness, likely due to increased lattice strain and defect formation induced by excessive dopant incorporation. Structural analysis confirmed that all films crystallize in the α-Ga_2_O_3_ phase with a corundum-type structure. Although the crystal phase remained unchanged upon doping, Al incorporation modified the preferred orientation, crystallite size, and lattice strain, indicating microstructural reorganization induced by Al^3+^ substitution within the α-Ga_2_O_3_ lattice. Optical measurements showed that all samples maintain high transparency (≈90%) in the visible region. A gradual widening of the optical bandgap from 4.82 eV (undoped) to 4.87 eV (3 vol % Al) was observed, confirming successful doping and demonstrating that the optical properties can be finely tuned through controlled dopant concentration. Electrical characterization revealed linear *I*–*V* behavior, indicating ohmic conduction. The resistance, determined using Ohm’s law, decreased markedly from 17.32 MΩ for undoped α-Ga_2_O_3_ to 0.47 MΩ for the 3 vol % Al-doped film. Despite this substantial improvement in conductivity, the resistance values remain within the megaohm range, indicating that the films retain their wide-bandgap semiconducting character.

Overall, the findings highlight that moderate Al incorporation, particularly at 2 vol % in the precursor solution, provides the best balance between improved surface morphology, structural quality, optical transparency, and enhanced electrical performance, making these films promising candidates for optoelectronic and UV photodetector applications.

## Experimental

Undoped and Al-doped α-Ga_2_O_3_ thin films (with 1, 2, and 3 vol % Al in the precursor solution) were deposited onto Si substrates (15 × 15 mm^2^) by the spray pyrolysis technique under an O_2_ gas flow. The precursor solution was sprayed onto the substrates at an injection rate of 1.2 cc/min, using a nozzle-to-substrate distance of 30 cm. Gallium chloride (GaCl_3_, 99.8% purity) was purchased from Sigma-Aldrich and used as the Ga precursor at a concentration of 0.25 M dissolved in ethanol (C_2_H_5_OH), and the solution was homogenized in an ultrasonic bath for 30 min at 60 °C. Aluminum chloride (AlCl_3_, 99.8% purity, Sigma-Aldrich) was used as the Al dopant precursor. A 0.25 M AlCl_3_ solution was prepared for the doping of the α-Ga_2_O_3_ thin films. The substrate temperature was maintained at 500 °C during deposition, and each sample was deposited for 5 min. After deposition, all samples were thermally annealed under vacuum at 400 °C for 1 h. A longer annealing time of 1 h was chosen, compared to the deposition time of 5 min, to promote structural relaxation and crystallization, while a lower annealing temperature was selected to preserve the film stoichiometry, reduce residual stress, and minimize unwanted diffusion processes and degradation of the film surface.

Surface morphology and elemental composition of the deposited films were investigated using a Hitachi SU8230 scanning electron microscope equipped with an energy-dispersive X-ray spectroscopy detector. The surface topography and roughness were characterized by atomic force microscopy using a Park Systems instrument operated in non-contact mode with silicon cantilevers (tip radius of approximately 6 nm and spring constant of approximately 37 N/m). The structural properties of the undoped and Al-doped α-Ga_2_O_3_ thin films were examined by X-ray diffraction using a Rigaku SmartLab X-ray diffractometer with Cu radiation (Kα_1_ = 1.540598 Å, Kα_2_ = 1.544426 Å, and Kβ_1_ = 1.39217 Å), operated at 45 kV and 200 mA. Optical transmittance spectra were recorded using a JASCO V-670 UV–vis–NIR spectrophotometer to evaluate the transparency of the films. The optical bandgap energy was determined from the transmittance data using the Tauc relation. The electrical properties of the films were investigated by current–voltage measurements using a source meter to measure the current under an applied voltage. The measurements were performed to evaluate the influence of Al doping on the electrical conductivity of the films.

## Data Availability

Data generated and analyzed during this study is available from the corresponding author upon reasonable request.
